# Demyelinating and Thrombotic Diseases of the Central Nervous System: Common Pathogenic and Triggering Factors

**DOI:** 10.3389/fneur.2015.00063

**Published:** 2015-03-24

**Authors:** Tatiana Koudriavtseva, Rosaria Renna, Domenico Plantone, Caterina Mainero

**Affiliations:** ^1^Neurology Unit, Multiple Sclerosis Center, Regina Elena National Cancer Institute, IFO, Rome, Italy; ^2^Athinoula A. Martinos Center for Biomedical Imaging, Massachusetts General Hospital, Boston, MA, USA; ^3^Harvard Medical School, Boston, MA, USA

**Keywords:** multiple sclerosis, acute disseminated encephalomyelitis, neuromyelitis optica, antiphospholipid syndrome, cerebral venous thrombosis, thrombosis

## Introduction

Demyelinating diseases of the central nervous system (CNS) affect prevalently young adults and represent the main cause of neurological disability after trauma in this population (1). Multiple sclerosis (MS), acute disseminated encephalomyelitis (ADEM), and neuromyelitis optica (NMO) are the most common inflammatory-demyelinating disorders of the CNS (1, 2).

Multiple sclerosis shares several features with antiphospholipid syndrome (APS) including the clinical presentation, the relapsing–remitting course, the higher incidence in females of childbearing age, and the presence of similar white matter (WM) lesions at MRI (3). Likewise, both neurological symptoms and MRI lesions may overlap in ADEM and in the initial presentation of APS (4). Therefore, MS, ADEM, and APS are part of the reciprocal differential diagnosis (2). APS represents also one of the main risk factors for cerebral venous thrombosis (CVT) (5), which is not usually included in the differential diagnosis of demyelinating diseases. However, several reports in literature have described an association between CVT and MS (6–9).

Although an accurate differential diagnosis is desirable for ensuring a more targeted therapy, the examination of the shared features between thrombotic and demyelinating diseases of the CNS would help to understand their common pathogenic mechanisms.

## Specificity and Similarities of Thrombotic and Demyelinating Diseases

### Cerebral venous thrombosis

Cerebral venous thrombosis of dural sinus and/or large veins is considered as a rare form of cerebrovascular disease (0.5–1% of all strokes) (5, 10, 11), though a previous pathological study found higher prevalence of CVT (9.3%) in 182 consecutive autopsies (12). Although firstly recognized as an infective disease, CVT is now considered as a non-septic condition (11). Infections, however, mainly parameningeal, are recognized as a common cause of CVT in children (up to 40% of cases) (5). Other predisposing factors include both acquired (APS, pregnancy, puerperium, oral contraceptives, surgery, head trauma, dehydratation, cancer, and parameningeal infections) and genetic (deficiency of antithrombin-III, protein C and S, factor V Leiden positivity) conditions (5). CVT can occur at any age but it is more common in young people: in the largest cohort study, it has been reported that 78% of cases occur in patients younger than 50 years (13). The diagnosis of CVT is challenging in routine practice because the clinical manifestations may mimic several other diseases and may be restricted to isolated headache in up to 25% of patients (5). No laboratory parameters are diagnostic of CVT or can rule out it, as, for instance, a normal d-dimer level. Brain MRI frequently shows non-specific lesions, such as hemorrhages, infarcts, edema, and diffuse brain swelling or may be normal in up to 25% of cases. Gradient-echo and susceptibility-weighted MR imaging may increase CVT diagnostic sensitivity, especially in the early thrombotic stages (5). The overall prognosis of CVT is better than that of arterial stroke, with complete recovery in about two-thirds of cases (11).

### Acute disseminated encephalomyelitis

Acute disseminated encephalomyelitis is a monophasic demyelinating disease of CNS, which typically ensues to viral or bacterial infections and vaccinations occurring prevalently in children during the winter and spring (4, 14, 15). The isolation of a specific pathogen is uncommon despite the high prevalence of many neurotropic pathogens in the general population. The clinical symptoms are frequently non-specific such as headaches, fever, and lethargy, while MRI detects widespread, multifocal, and extensive white and deep gray matter lesions with poorly defined margins, and sometimes large, swollen, thoracic cord lesions, completely resolving in up to half of the cases. There is a report of even normal MRI in ADEM (16). Furthermore, in acute hemorrhagic leukoencephalitis, a severe and rare variant of ADEM, large WM lesions with edema, mass effect and even obvious hemorrhages are detected on MRI (1). Although the MRI features of ADEM have not been clearly established, a recent study demonstrated a prevalently vasogenic edema (17).

Despite CVT and ADEM are not usually part of the reciprocal differential diagnosis, the MRI characteristics of ADEM as well as its clinical presentation and evolution appear more similar to CVT rather than to MS. It should be also highlighted that large thoracic lesions reported in ADEM are a hallmark of APS in differential diagnosis with MS (3).

### Antiphospholipid syndrome

The diagnostic criteria of APS include arterial/venous thrombosis and/or fetal loss, and the persistence of specific antiphospholipid antibodies (aPL) at medium or high titer for at least 12 weeks (18). APS might be associated with infections (19, 20).

Antiphospholipid antibodies are not exclusive of APS and may be found less steadily and/or in low titers in infections (21), in CVT (5), and in several autoimmune diseases including MS (22–25) and NMO spectrum disorders (NMOSD) (26). Reactivity for aPL and other autoantibodies has been reported more frequently in NMOSD compared to MS (26, 27), confirming the already known higher occurrence of aPL in “neuromyelitic” type of MS (28).

### Neuromyelitis optica

Neuromyelitis optica is a more severe demyelinating disorder compared to MS. It is characterized by optic neuritis, longitudinally extensive myelitis (three or more spinal segments on MRI), and autoantibody positivity against the water channel aquaporin-4 (2, 29). Large spinal cord lesions, similar to those of APS and ADEM, represent one of the diagnostic criteria for NMO. Recently, anti-cardiolipin antibodies in NMO patients have been associated with greater antithrombin-III activity and d-dimer levels, a product of fibrin degradation, suggesting the involvement of thrombotic pathogenic mechanisms in this disorder (26, 30). Moreover, several studies supported the association between NMO and various infections (31–33).

### Multiple sclerosis

Multiple sclerosis is a chronic disorder of the CNS pathologically characterized by inflammation, demyelination, and neurodegeneration. MS usually begins with a relapsing–remitting course manifesting with relapses, commonly followed by a secondary progressive phase characterized by steady accumulation of disability. As mentioned before, aPL can be detectable in MS, and their reactivity appears to be related to the severity of the MS stage: aPL reactivity is higher in secondary progressive MS than in relapsing–remitting MS, and particularly during the relapses (up to 80%) (22–25). Interestingly, aPL reactivity decreases over few months after relapse. Furthermore, aPL positive relapsing–remitting MS patients develop more severe clinical and MRI disease progression over a 3-year follow-up compared to aPL negative MS cases (34).

Multiple sclerosis is associated with an increased risk of venous thromboembolism (35). CVT has been also reported in MS, being generally attributed to lumbar puncture and/or to the effects of methylprednisolone therapy, but in some cases, it was not associated with any predisposing state (6–9). Furthermore, some conditions that favor thrombosis such as cranial radiation, CNS trauma, and puerperium may promote MS reactivation (36). Also stress, producing significant hemoconcentration and pro-thrombotic blood changes (37), might foster MS relapse. However, the main risk factors for MS re-exacerbations are represented by infections, mostly viral (36). Viruses are considered to be the principal environmental factors associated with MS as suggested by epidemiological and genetic studies (38), as well as by the lifelong presence of oligoclonal IgG in the cerebrospinal fluid in the majority of MS patients (39).

## Discussion

The close clinical and radiological similarities between inflammatory-demyelinating and thrombotic diseases of the CNS suggest the presence of common underlying events. There is a striking incongruence regarding the prevalence of CVT, commonly acknowledged to represent 0.5–1% of all strokes, but found in the 9.3% of consecutive autopsies. This apparent incongruence can be partially explained not only by the difficulty of CVT clinical diagnosis but also by the lower occurrence of CVT of dural sinus or large veins compared to the thrombosis of smaller veins occurring, for example, in APS. Thus, on the basis of similar clinical and MRI features of CVT and ADEM, it can be hypothesized that inflammatory-thrombotic events take part in the pathophysiology of ADEM, even if to a lesser extent. In the same way, the characteristics of spinal cord lesions as well as a high frequency of aPL associated with increased coagulation indicators may suggest the presence of inflammatory-thrombotic phenomena in NMO. Indeed, further MR-based studies assessing brain and spinal cord venous blood dynamics are needed to confirm such hypothesis.

Similarly, the association of MS with an increased risk of venous thromboembolism reported in epidemiological studies, as well as the correlation between MS exacerbations and pro-thrombotic factors including aPL positivity, would suggest that MS pathogenic mechanisms may, at least in part, involve thrombotic processes (40). Moreover, in experimental allergic encephalomyelitis (EAE), fibrin deposition precedes and regulates the inflammatory demyelination, while its genetic or pharmacologic depletion ameliorates both clinical symptoms and inflammatory response (41). Early perivascular microglial clustering is blocked by anticoagulant treatment or by genetic depletion of fibrinogen in EAE (42). Thrombin, which has numerous hormone-like functions affecting microglia and astrocytes, was proposed as a therapeutic target in MS (43). On the other hand, the concept of thrombo-inflammation was recently introduced since ischemic stroke has been defined as a thrombo-inflammatory disease (44). Indeed, both innate and adaptive immunity are involved during all stages of stroke (45). The inhibition of plasma kallikrein, a key constituent of the proinflammatory contact-kinin system, is effective in wild-type mice up to 3 h post stroke (46). In fact, the contact-kinin system, representing an interface between inflammatory and thrombotic systems, was found activated in different neurological diseases, such as in traumatic brain injury showing a microvascular thrombosis along with edema and immune cell infiltration (47). The data reported in this work would suggest that the concept of thrombo-inflammation is also appropriate for the inflammatory-demyelinating diseases, albeit with quantitatively varying expression.

Inflammation and coagulation represent the main components of innate immunity, an universal immediate defense against infections, which stimulates and modulates the adaptive one. Thrombotic events occur when coagulation processes prevail over the natural anticoagulant system (48). Therefore, the presence of thrombosis should indicate the occurrence of intense and/or prolonged inflammatory-thrombotic processes. This could take place in the course of subclinical chronic infections prevalently in individuals with a reduced anticoagulant and/or anti-oxidative capacity, resulting in their individual susceptibility to these diseases. All disorders described above occur prevalently in the young age, characterized by a first contact with external pathogens, and are closely linked with concomitant or immediately preceding clinical infections. It is more realistic to assume that a massive and steady response of innate immunity is triggered directly by the pathogen rather than by an indirect autoimmune reaction. Even autoantibodies including aPL may be an expression of a reaction against the self-components damaged during infections or injury similarly to physiological process of elimination of aged cells and proteins.

In conclusion, we speculate that demyelinating and thrombotic diseases of the CNS share widespread occurrence of inflammatory-thrombotic processes. The triggering factors underlying these processes are still unknown. Inflammatory and coagulant pathways are closely linked during activation of the innate immune system. Hence, it is possible that common pathogenic factors, including subclinical chronic infections, exert their role at the level of the innate immune system in genetically or environmentally induced susceptible individuals. This viewpoint does not aim to belittle the importance of specific characteristics of these diseases, which could be related to different causal pathogens together with concomitant and genetic factors, but it is aimed to find their reciprocal factual connections in order to better control them and develop more appropriate therapeutic strategies.

## Conflict of Interest Statement

The authors declare that the research was conducted in the absence of any commercial or financial relationships that could be construed as a potential conflict of interest.
